# Systematic literature review of pharmacists in general practice in supporting the implementation of shared care agreements in primary care

**DOI:** 10.1186/s13643-022-01933-4

**Published:** 2022-05-11

**Authors:** Naveed Iqbal, Chi Huynh, Ian Maidment

**Affiliations:** Aston Pharmacy School, College of Health and Life Sciences, Aston University, Aston Triangle, Birmingham, B4 7ET UK

**Keywords:** Shared care, Pharmacists, Long-term conditions, Systematic review, integrated care, Physicians, Primary Care, Seamless care

## Abstract

**Background:**

Rising demand for healthcare continues to impact all sectors of the health service. As a result of the growing ageing population and the burden of chronic disease, healthcare has become more complex, and the need for more efficient management of specialist medication across the healthcare interface is of paramount importance. With the rising number of pharmacists working in primary care in clinical roles, is this a role that pharmacists could support to ensure the successful execution of shared care agreement (SCA) in primary care for these patients?

**Aim of the review:**

Systematic review to identify activities and assess the interventions provided by pharmacists in primary care on SCA provision and how it affects health-related quality of life (HRQoL) for patients.

**Method:**

Primary studies in English which tested the intervention or obtained views of stakeholders related to pharmacist input to shared care agreement within primary care were included. The following electronic databases were systematically searched from the date of inception to November 2021: AMED®, CINAHL®, Cochrane Database of Systematic Reviews (CDSR), EMBASE®, EMCARE®, Google Scholar, HMIC®, MEDLINE®, PsycINFO®, Scopus and Web of Science®. Grey literature sources were also searched. The search was adapted according to the respective database-specific search tools. It was searched using a combination of Medical Subject Heading terms (MeSH), free-text search terms and Boolean operators.

**Results:**

A total of 5244 titles/abstracts were screened after duplicates were removed, and 64 full articles were assessed for eligibility. On examination of full text, no studies met the inclusion criteria for this review.

**Conclusion:**

This review highlights the need for further research to evaluate how pharmacists in general practice can support the safe and effective integration of specialist medication in primary care with the use of SCA.

**Systematic review registration:**

NIHR PROSPERO No: 2020 CRD42020165363.

**Supplementary Information:**

The online version contains supplementary material available at 10.1186/s13643-022-01933-4.

## Introduction

Healthcare systems in the world are facing significant challenges as a result of severe funding pressure, a growing ageing population, societal changes, rising demand and a limited supply of some healthcare professional groups [1–3]. This is compounded by the increasing prevalence of long-term conditions (LTC), in particular, people having two or more conditions which are being supported by different parts of the healthcare system [4]. The UK is home to the National Health Service (NHS), one of the largest healthcare systems in the world. In the UK, LTCs account for 70% of the NHS healthcare budget [4]. With the continued demands in the NHS, the current models of dealing with long-term conditions are not sustainable; the need to innovate in order to continue to deliver world-class healthcare outcomes within a limited financial envelope is critical [5]. Care needs to be provided in the right place and at the right time to ensure that the healthcare system meets the current and future needs of a nation’s healthcare provision [6]. In response to this new normal, care must not be fragmented between healthcare systems, e.g. pre-hospital, hospital and specialist care [7].

This is of particular importance in countries where healthcare systems are well developed where care needs to move seamlessly from the *gatekeeping* primary care systems to the hospital and specialist services for the benefit of patient care [8]. Effective communication and cooperation between primary and secondary care are critical to making the best use of limited resources [9] and to ensure the patient receives high-quality joined-up care [8, 10, 11]. A tool that has been used in the literature to support effective integrated care between primary and secondary care is the shared care agreement (SCA) [12–14]. The SCA was designed to provide a framework for the seamless sharing of care and to facilitate the passage of hospital-prescribed medication or a consultant-managed patient into primary care.

The term shared care agreement has various definitions, none of which is universally agreed on [14–18]. The primary description to describe shared care was by Hickman et al. in their seminal paper on the taxonomy of shared care in 1994 [15]. The original definition of shared care described shared care as *The joint participation of general practitioners and hospital consultants in the planned delivery of care for patients with chronic inflammatory musculoskeletal disorders, informed by an enhanced information exchange over and above the routine clinic, discharge and referral letters* [15]. A more recent evolved definition describes these arrangements as *the joint participation of primary and speciality care practitioners in the planned delivery of care for patients with a chronic condition, informed by enhanced information exchange, over and above routine discharge and referral notices* [16]. This evolved definition takes into account the changes in primary care since the primary definition was described.

Since the introduction of an executive letter EL (91) 127 by the NHS in 1991 described SCA to support prescribing between hospitals and GPs [19], several problems have marred the integration of SCA into primary care, and in 2017, the British Medical Association (BMA) stated that shared care is still not working effectively [20]. General practitioners have expressed concern about poor communication between primary and secondary care, lack of follow-up and monitoring and the medico-legal responsibilities for the prescriber when they accept shared care [16, 21–23]. After 30 years, EL (91) 127 has been superseded by the new national guidance released in 2018 for England [24]. The guidance was designed to overcome the challenges of shared care that have been exhibited in the healthcare system over the last 30 years [21]. The document has described a role for the pharmacist in general practice in supporting joint working and collaboration to ensure that primary care prescribers have access to information on new or less familiar medicines and how they can support the introduction of medicines into primary care [21]. Evidence indicates that pharmacists have a significant role in medicine optimisation and improving safe and effective medication use in primary care [25]. In addition, the literature has shown that pharmacists in general practice are increasingly playing a central role in managing medicines [26], such as setting up systems for safe monitoring and prescribing high-risk medicines such as direct oral anticoagulants, lithium, non-steroidal anti-inflammatory drugs (NSAIDs) prescribing and medicine safety requirement in general practice. With the support of government initiatives to increase the number of pharmacists in general practice to support new models of care, the pharmacist could be seen as a vital component in bridging the transfer of care from secondary to primary care settings [4, 27, 28]. This paper aims to review the literature on the role of a pharmacist in general practice with regard to SCA support, their roles and identify the potential benefits, barriers and facilitators to their potential integral role.

## Aim of the review

This study aimed to identify activities and assess the interventions provided by pharmacists in primary care on SCA provision. The specific objectives were to determine the following:

- The types of interventions/activities being provided by pharmacists in supporting SCA in a general practice setting
- The effectiveness of these interventions/activities on health-related quality of life (HRQoL) for patients, to consider the impact of clinical pharmacist supporting shared care agreement in a general practice setting.

## Method

### Study registration

The review protocol was registered with the International Prospective Register for Systematic Review (PROSPERO database; registration number CRD42020165363). The review was guided by the recommendations of the Cochrane Handbook for Systematic Reviews of Interventions [29]. The reporting of the review complies with the Preferred Reporting Items for Systematic Review and Meta-Analysis (PRISMA) statement and checklist (refer to supplementary information S2 and S3) [30].

### Eligibility criteria for study inclusion

The search was focused on locating studies eligible for inclusion or excluded based on the criteria below.

#### Inclusion criteria

The following are the inclusion criteria:

1. Only primary studies (qualitative, quantitative and mixed studies)
2. Studies which have tested an intervention and/or have obtained views of stakeholders (pharmacists, GPs, medical specialists, practice staff and patients) related to SCA in the primary care setting
3. Studies in which a pharmacist has input within a primary care setting to provide non-dispensing care

#### Exclusion criteria

The following are the exclusion criteria:

1. Studies in which the intervention has been provided only in secondary or tertiary care settings (hospitals, specialist clinics and national and regional specialist centre)
2. Studies written in a language other than English
3. Studies presented as editorials, protocols and commentaries

### Information sources and search strategy

Specific search strategies were developed with expert information specialists and included broad and narrow, free-text and thesaurus-based terms. The term pharmacist was used as a general term to allow greater scope to find multiple roles provided by pharmacists, which included prescribers. Boolean operators and truncation were used to ensure we maximised our search strategy. The following eleven databases were systematically searched from date of inception to November 2021: Allied and Complementary Medicine Database (AMED®) (1985 to 07.11.2021) Platform: Ovid®; Cumulative Index to Nursing and Allied Health Literature (CINAHL®) (1950 to 07.11.2021) Platform: EBSCO®; Cochrane Database of Systematic Reviews (CDSR) (accessed on 07.11.2021) Platform: Wiley® online library; Excerpta Medica database (EMBASE®) (1974 to 07.11.2021) Platform: Ovid®; EMCARE® (1995 to 07.11.2021) Platform: Ovid®; Google Scholar (accessed on 07.11.2021) Platform: Google UK®; Healthcare Management Information Consortium (HMIC®) (1979 to November 2021) Platform: Ovid®; Medical Literature Analysis and Retrieval System Online (MEDLINE®) (1946 to 07.11.2021) Platform: Ovid®; PsycINFO®, Psychology and Behavioural Sciences Collection, Health Business Elite, Biomedica Reference Collection: Comprehensive Library, Information Science & Technology Abstracts (1967 to 07.11.2021) Platform: EBSCOhost®; and Scopus (2004 to 07.11.2021) Platform: Elsevier, Web of Science® Core Collection (1970-07.11.2021) Platform: Clarivate Analytics®. The search was adapted according to the respective database-specific search tools. It was searched using a combination of Medical Subject Heading terms (MeSH) where available, free-text search terms and Boolean operators. Refer to supplementary information S1 ‘Search terms’ for the specific detail of the search used for each database. Search results in languages other than English were noted, but for practical reasons, only search results in English or translated into English were included in this review. In an effort to identify unpublished studies, a search of grey literature was performed (http://www.opengrey.eu/ on 07.11.2021) to identify studies not indexed in the databases listed above. The term grey literature in this paper refers to sources used to describe a wide range of information produced outside of traditional publishing and distribution channels and which is often not well represented in indexing databases.

### Data collection and analysis

All references from database search were downloaded into EndNote® X8.2 [31] reference manager, which was used to collate and remove duplicate records, to screen titles and abstracts and to store the full text of retrieved studies. Citations from OpenGrey could not be uploaded to EndNote® reference manager and therefore were uploaded to a Microsoft Excel® 2016 spreadsheet. Duplicate citations were removed by the automatic de-duplicating option in EndNote®X8.2 and were supplemented by hand-searching. Two researchers (NI and CH) examined the titles and abstracts of all eligible articles according to the inclusion and exclusion criteria listed above. References to be screened were allocated into groups and was divided into ‘include’, ‘exclude’ and ‘potential’ groupsets. The full-text articles of any abstracts classified as potentially meeting the inclusion criteria were retrieved and analysed independently by two authors (NI and CH) against the predefined inclusion and exclusion criteria with differences between reviewers resolved by consensus. The principal authors of all included papers were contacted to explore the potential for any studies considered vital to them that may have been missed in the search strategy. A data extraction form from the Cochrane collaboration was utilised to extract data from eligible papers [32]. Raw data from quantitative studies were extracted onto Microsoft Excel® 2016 spreadsheet. If data could not be pooled for meta-analysis, the plan was to undertake a narrative synthesis of results. The qualitative data synthesis methodology was decided upon after the quantity, quality, conceptual richness, and contextual thickness of the qualitative studies were determined. The intended results and data synthesis were to be conducted by authors NI and CH independently to assure the data extraction integrity.

The Mixed Method Appraisal Tool (MMAT) 2018 version was used to appraise and describe the methodological quality of included quantitative, qualitative and mixed-method studies. A pilot test of two articles was conducted to ensure consistent interpretation between the two authors (NI and CH). Discrepancies were resolved through discussion and consensus with the research team. If further information was required to appraise a particular study, an attempt was made to contact the authors by phone or email. Quality scores will be calculated using the MMAT tool. However, this did not solely determine if studies were of “low” or “high” quality, as a descriptive summary using MMAT criteria was considered [33]. If a study received a low score, it was compared with those with a higher score (higher quality studies) to consider if this modifies the outcome and interpretation of our synthesis.

## Results

The database search yielded 7727 citations. After duplicates were removed, the database search identified 3908 citations. Based on the title and abstract information, 3844 citations were ‘excluded’, leaving 64 citations identified as ‘potentially relevant’ articles requiring a full-text article text review. In addition to the 3908 citations that were yielded by the databases, 1336 additional citations were retrieved from http://www.opengrey.eu/ and were screened separately as they could not be uploaded onto EndNote® X8.2 [31] reference manager. After screening, no pertinent articles that met the inclusion and exclusion criteria were extracted from grey literature sources.

**Fig. 1:**
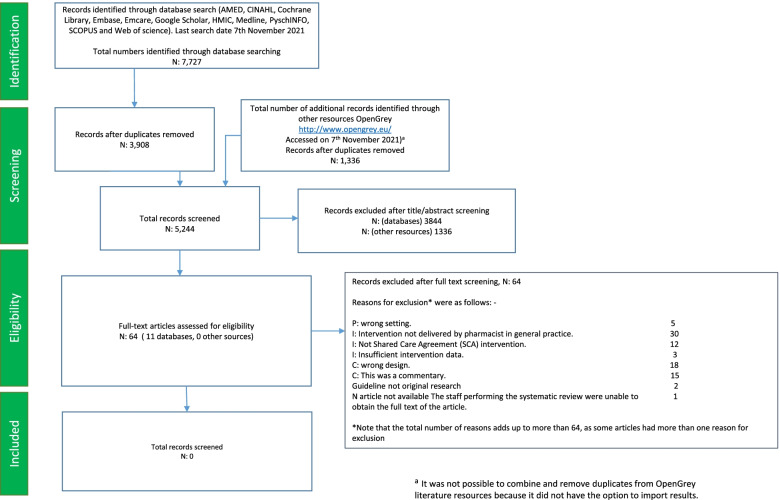
Preferred Reporting Items for Systematic Reviews and Meta-Analysis (PRISMA) flow chart

On the examination of the full text of the 64 studies in the ‘potentially relevant’ category, no publications addressing evidence to identify activities and to assess interventions provided by pharmacists in primary care on SCA provision were identified (see Table 1, which includes a tabulated list of the 64 excluded studies along with reasons for exclusion, with some articles having more than one reason for exclusion). Out of the 64 excluded studies, one study (a conference proceeding) could not be found, and an attempt was made to contact the author to obtain further information (full set of results). The author did not respond; hence, this article was excluded from our review as the reviewers could not assess the eligibility based on the information from the abstract. The Preferred Reporting Items for Systematic Reviews and Meta-Analysis (PRISMA) flow chart (Fig. 1) shows the identification, screening and selection of papers for this review. There were no included studies for which to assess the risk of bias or to apply for evidence synthesis.

**Table 1:** List of excluded studies along with reasons for exclusion

Number	Authors	Country of origin	Study title	Aim of the study	Reason for exclusion
1	Adams B (2015) [34]	England	NHS Alliance: the evolution of primary care	This paper highlighted the NHS Alliance conference, which focused on pharmacist integration and GP work burden.	C: This was a commentary.
2	Agyapong, V. I., et al. (2011) [35]	Ireland	Shared care between specialized psychiatric services and primary care: the experiences and expectations of consultant psychiatrists in Ireland	The article explores the views of consultant psychiatrists in Ireland on shared care between specialized psychiatric services and primary care for patients with mental health difficulties.	I: Intervention not delivered by a pharmacist in general practice. The intervention was not delivered by the person specified in the review criteria.
3	Al-Alawi, K., et al. (2019) [36]	Oman	Care providers’ perceptions towards challenges and opportunities for service improvement at diabetes management clinics in public primary health care in Muscat, Oman: a qualitative study	The article explores the challenges and discusses opportunities for improvement of diabetes management clinics in primary healthcare centres in Oman.	I: Intervention not delivered by a pharmacist in general practice. The intervention was not delivered by the person specified in the review criteria. I: Not a shared care agreement (SCA) intervention. The study used the wrong model of intervention.
4	Alhabib, S., et al. (2016) [37]	Saudi Arabia	An evolving role of clinical pharmacists in managing diabetes: evidence from the literature	This article is a narrative review of the evidence of the role of clinical pharmacists in managing diabetic patients.	I: Not a shared care agreement (SCA) intervention. The study used the wrong model of intervention.
5	Ali, S., et al. (2013) [38]	USA	Psychiatric providers’ willingness to participate in shared decision-making (SDM) when prescribing psychotropic medications	This article aims to determine the psychiatric providers’ willingness to engage in SDM and factors that influence willingness.	I: Intervention not delivered by a pharmacist in general practice. The intervention was not delivered by the person specified in the review criteria. I: Not a shared care agreement (SCA) intervention. The study used the wrong model of intervention.
6	Aljumah, K. and M. A. Hassali (2015) [39]	Saudi Arabia	Impact of pharmacist intervention on adherence and measurable patient outcomes among depressed patients: a randomised controlled study	This article evaluates the effectiveness of shared decision-making on pharmacist intervention for improving adherence and patient outcomes, compared with usual care.	I: Not a shared care agreement (SCA) intervention. The study used the wrong model of intervention. C: Wrong design. The design of the study was not the one desired.
7	Almunef, M., et al. (2019) [40]	England	Management of chronic illness in young people aged 10–24 years: a systematic review to explore the role of primary care pharmacists	The article examines the role of primary care pharmacists in the management of chronic illnesses in young people aged 10–24 years.	I: Insufficient intervention data. Intervention was not described in enough detail to determine the study should be included. C: Wrong design. The design of the study was not the one desired.
8	Alshehri et al. (2021) [41]	England	Evaluating the role and integration of general practice pharmacists in England: a cross-sectional study	The paper aims to assess the role performed by GP pharmacists and their integration into practice exploring facilitators and barriers to integration across England.	I: Insufficient intervention data. Intervention was not described in enough detail to determine the study should be included. I: Not a shared care agreement (SCA) intervention. The study used the wrong model of intervention.
9	Anderson, K., et al. (2015) [42]	Australia	Polypharmacy, deprescribing and shared decision-making in primary care: the role of the accredited pharmacist	Commentary: The article discusses the role of the pharmacist in supporting deprescribing and reducing polypharmacy.	C: This was a commentary.
10	Anonymous (1994) [43]	England	General practitioner/pharmacy interface a local initiative	Commentary: A primary care development officer describes how communication between general practitioners and pharmacists has been improved in Sunderland.	C: This was a commentary.
11	Anonymous (1999) [44]	England	The role of pharmacists in primary care groups (PCGs): a strategic approach	Conference: Examined issues surrounding the relevance and role of pharmacists within the new NHS structures, with a particular emphasis on how pharmacists could contribute to the work of PCGs.	C: This was a commentary.
12	Anonymous (1995) [45]	England	Pharmacy in a new age: the role of government	Commentary: This paper considers important factors shaping the future of pharmacy.	C: This was a commentary.
13	Anonymous (2003) [46]	England	Shared care addiction scheme starts	Commentary: The paper discusses a seamless addiction service involving hospital and community pharmacists.	C: This was a commentary.
14	Ashcroft, D. M., et al. (1998) [47]	England	Shared care: a study of patients’ experiences with erythropoietin	The article evaluates the impact of a shared care scheme on patients receiving erythropoietin treatment.	I: Intervention not delivered by a pharmacist in general practice. The intervention was not delivered by the person specified in the review criteria.
15	Bains, S., et al. (2021) [48]	England	The pharmacist-led accelerated transfer of patients to shared care for the monitoring and prescribing of immunomodulatory therapy during COVID-19.	Conference abstract: The conference abstract discusses the use of secondary care pharmacist in supporting transfer of medication into primary care.	I: Intervention not delivered by a pharmacist in general practice. The intervention was not delivered by the person specified in the review criteria. C: Wrong design. The design of the study was not the one desired.
16	Bajramovic, J., et al. (2004) [49]	Australia	Perceptions around concordance—focus groups and semi-structured interviews conducted with consumers, pharmacists and general practitioners	The article explores the beliefs and expectations of general practitioners, consumers and pharmacists in relation to concordance.	C: Wrong design. The design of the study was not the one desired.
17	Barnes, E., et al. (2017) [50]	England	New roles for clinical pharmacists in general practice.	Commentary: The paper discusses the NHS England scheme to fund, recruit and employ more clinical pharmacists in GP practices.	C: This was a commentary.
18	Bellingham, C. (2004) [51]	England	How to improve medicines management at the primary/secondary care interface	Commentary: The paper discusses medicines management at the interface.	C: This was a commentary.
19	Berendsen, A. J., et al. (2009) [52]	Netherlands	Transition of care: experiences and preferences of patients across the primary/secondary interface—a qualitative study.	The article explores the transition of care at the primary–secondary interface with reference to the impact of patients’ ability to make choices about their secondary care providers.	I: Not a shared care agreement (SCA) intervention. The study used the wrong model of intervention. C: Wrong design. The design of the study was not the one desired.
20	Bojke, C., et al. (2010) [53]	England	Cost-effectiveness of shared pharmaceutical care for older patients: RESPECT trial findings	The article evaluates the cost-effectiveness of shared pharmaceutical care for older people compared to usual care.	I: Not a shared care agreement (SCA) intervention. The study used the wrong model of intervention. C: Wrong design. The design of the study was not the one desired.
21	British National Association of Health Authorities and Trusts (1994) [54]	England	NAHAT Update: asthma care—the challenge ahead	Commentary: This paper examines the challenges which the treatment and management of asthma for all sectors of the health service.	C: This was a commentary.
22	Cain, R. M. (2006) [55]	USA	The physician-pharmacist interface in the clinical practice of pharmacy	Commentary: This paper deals with healthcare interface and how this may influence the overall practice of clinical pharmacy.	C: This was a commentary.
23	Carrington, I. and J. McAloon (2018) [56]	Northern Ireland	Why shared-care arrangements for prescribing in attention deficit hyperactivity disorder may not be accepted	The article explores the reasons for the failure of uptake of shared-care arrangements for prescribing in attention deficit hyperactivity disorder.	I: Intervention not delivered by a pharmacist in general practice. The intervention was not delivered by the person specified in the review criteria.
24	Chana, N., et al. (2017) [57]	England	Improving specialist drug prescribing in primary care using task and error analysis: an observational study	The article explores how clinical decision support systems can support GPs in prescribing specialist drugs using a task and error analysis.	I: Intervention not delivered by a pharmacist in general practice. The intervention was not delivered by the person specified in the review criteria.
25	Chartrand, M., et al. (2013) [58]	Canada	Implementation and evaluation of pharmacy services through a practice-based research network (PBRN)	The article discusses how to develop a web-based PBRN and assess the feasibility of this intervention.	I: Not a shared care agreement (SCA) intervention. The study used the wrong model of intervention.
26	Cox, W. M. (2002) [59]	Wales	Evaluation of a shared-care program for methadone treatment of drug abuse: an international perspective	This article evaluates a North Wales Shared-Care Program for methadone for the treatment of drug abuse.	I: Intervention not delivered by a pharmacist in general practice. The intervention was not delivered by the person specified in the review criteria. I: Not a shared care agreement (SCA) intervention. The study used the wrong model of intervention.
27	Crowe, S., et al. (2009) [60]	England	The prescribing of specialist medicines: what factors influence GPs’ decision making?	The article explores the factors which influence GPs’ decision-making process when requested to prescribe a specialist drug.	I: Intervention not delivered by a pharmacist in general practice. The intervention was not delivered by the person specified in the review criteria.
28	Crowe, S., et al. (2010) [61]	England	Shared care arrangements for specialist drugs in the UK: the challenges facing GP adherence	The article explores the challenges facing GPs’ adherence to shared care arrangements for specialist drugs.	I: Intervention not delivered by a pharmacist in general practice. The intervention was not delivered by the person specified in the review criteria.
29	Duggan, C., et al. (2001) [62]	England	Shared care in the UK: failings of the past and lessons for the future	To article explores and evaluates the implementation of shared care in the UK.	I: Intervention not delivered by a pharmacist in general practice. The intervention was not delivered by the person specified in the review criteria.
30	Fearne, J., et al. (2018) [63]	Malta	Development and evaluation of shared paediatric pharmaceutical care plan	The article explored the development of a shared paediatric pharmaceutical care template aimed at improving communication between pharmacists across different care settings.	I: Intervention not delivered by a pharmacist in general practice. The intervention was not delivered by the person specified in the review criteria. I: Not a shared care agreement (SCA) intervention. The study used the wrong model of intervention.
31	Finch, E. and C. Ford (2002) [64]	England	Shared care at the primary and secondary interface: GPs and specialist drug services	Book: This book chapter describes shared care of drug users in the UK.	C: This was a commentary.
32	Grixti, D., et al. (2014) [65]	Malta	Development of shared care guidelines in rheumatology	A poster presentation on the development of shared care guidelines for rheumatology drugs. With the intent of providing seamless care between primary and secondary care settings.	I: Intervention not delivered by a pharmacist in general practice. The intervention was not delivered by the person specified in the review criteria.
33	Gu, Y., et al. (2012) [66]	New Zealand	An Innovative Approach to Shared Care–New Zealand Pilot Study of a Technology-enabled National Shared Care Planning Programme	The article describes progress and lessons learned from the New Zealand National Shared Care Planning Programme.	I: Not a shared care agreement (SCA) intervention. The study used the wrong model of intervention.
34	James, O., et al. (2020) [67]	Ireland	Pharmacists in general practice: a qualitative process evaluation of the General Practice Pharmacist (GPP) study	The article explores the implementation of The General Practice Pharmacist (GPP) intervention and the experiences of study participants and lessons for future implementation.	I: Not a shared care agreement (SCA) intervention. The study used the wrong model of intervention.
35	Johnson, C. (2018) [68]	USA	Adult attention deficit and hyperactivity disorder (ADHD) clinic: a collaboration between psychiatry, primary care and pharmacy to improve access, care experience and affordability	The article discusses a collaborative, team-based adult ADHD service to improve the care experience.	I: Intervention not delivered by a pharmacist in general practice. The intervention was not delivered by the person specified in the review criteria.
36	Jones, B. W. and W. Clark (2003) [69]	England	Shared care agreements: how to overcome the blank page	This article describes how prescribing problems that occur across the primary-secondary care interface are being tackled by pharmacists and others in the West Midlands.	P: Wrong setting. The research setting was not correct. C: Wrong design. The design of the study was not the one desired.
37	Jones, C., et al. (2017) [70]	England	Update on the introduction of dose tapering to modernize and improve the biologics service in a district general hospital	Poster presentation: The poster presentation discusses the introduction of a departmental dose tapering protocol for patients with Rheumatoid arthritis.	P: Wrong setting. The research setting was not correct. I: Intervention not delivered by a pharmacist in general practice. The intervention was not delivered by the person specified in the review criteria.
38	Jones, E. and O. A. Cuevas (2018) [71]	England	An audit on the use and monitoring of azathioprine (AZA) in a paediatric gastroenterology centre. Could NHS England via specialist commissioning rules (NHS-E-SPR) be affecting quality of care?	Poster presentation: An audit of gastroenterology centre’s adherence to the British Society of Gastroenterology Hepatology and Nutrition (BSPGHAN) guideline.	P: Wrong setting. The research setting was not correct. I: Intervention not delivered by a pharmacist in general practice. The intervention was not delivered by the person specified in the review criteria.
39	Lloyd, L. A., et al. (2009) [72]	England	An audit of methotrexate monitoring in primary care as part of a shared care agreement	Conference abstract: The conference abstract discusses and audit of methotrexate.	I: Insufficient intervention data. Intervention was not described in enough detail to determine the study should be included.
40	MacLellan, J., et al. (2017) [73]	England	Shared care: How can we do it? Findings from the British HIV Association (BHIVA) Primary Care Project	Report: Report by BHIVA on Commissioning and delivery of high-quality healthcare for people with HIV between primary and specialist care across the life course.	C: Wwrong design. The design of the study was not the one desired. I: Intervention not delivered by a pharmacist in general practice. The intervention was not delivered by the person specified in the review criteria.
41	Mercer, K., et al. (2018) [74]	Canada	Physician and pharmacist medication decision-making in the time of electronic health records: mixed-methods study	The article examines how physicians and pharmacists understand and communicate patient-focused medication information using electronic health records.	I: Intervention not delivered by a pharmacist in general practice. The intervention was not delivered by the person specified in the review criteria. C: Wrong design. The design of the study was not the one desired.
42	Mercer, K., et al. (2020) [75]	Canada	“My pharmacist”: creating and maintaining relationship between physicians and pharmacists in primary care settings	The article examines how pharmacists and primary care physicians communicate with each other and maintain relationships.	C: Wrong design. The design of the study was not the one desired.
43	Morakinyo, J. (2017) [76]	England	Shared care guideline for the use of methylphenidate, dexamfetamine, lisdexamfetamine dimesylate & atomoxetine for the management of attention deficit hyperactivity disorder (ADHD) in adults (18–64 years).	An NHS document provides information allowing patients with ADHD to be managed safely via the transfer of prescribing across the primary and secondary care interface.	Guideline not original research.
44	Mousa, Y., et al. (2009) [77]	England	Investigating the potential for improved management of patients with long term conditions through shared-care protocols	This article evaluates the need and satisfaction of GPs and community pharmacists within the area with these guidelines.	I: Intervention not delivered by a pharmacist in general practice. The intervention was not delivered by the person specified in the review criteria.
45	NICE (2016) [78]	England	Transition between inpatient hospital settings and community or care home settings for adults with social care needs. National Institute for Health and Care Excellence	This guideline covers the transition between inpatient hospital settings and community or care homes for adults with social care needs.	Guideline not original research.
46	Nkansah, N., et al. (2010) [79]	International (Systematic Review: Cochrane Review). Authors based in the USA	Effect of outpatient pharmacists’ non-dispensing roles on patient outcomes and prescribing patterns	The article discusses outpatient pharmacists’ non-dispensing roles on patient and health professional outcomes.	C: Wrong design. The design of the study was not the one desired.
47	O’Halloran, K. A. (2016) [80]	England	Developing integrated care teams across the North West London System	Conference abstract on developing integrated care system in London.	C: This was a commentary.
48	Petty, D. (2019) [81]	England	Clinical pharmacist roles in primary care networks (PCN)	This article discusses the roles that will be expected of clinical pharmacists within PCNs and how these roles are likely to develop in the future.	C: This was a commentary.
49	Murphy, K (2018) [82]	Australia	Clozapine, concomitant medications and consumers: assessing the accuracy of medication records and the lived experience of people prescribed clozapine under shared care arrangements	This thesis discusses interventions to optimise access to accurate medication information, and communication pathways between stakeholders and consumers of clozapine shared care service.	I: Intervention not delivered by a pharmacist in general practice. The intervention was not delivered by the person specified in the review criteria.
50	Richmond, S., et al. (2010) [83]	England	Effectiveness of shared pharmaceutical care for older patients: RESPECT trial findings	The article evaluates the effectiveness of pharmaceutical care for older people, shared between GPs and community pharmacists in the UK, relative to usual care.	I: Intervention not delivered by a pharmacist in general practice. The intervention was not delivered by the person specified in the review criteria. C: Wrong design. The design of the study was not the one desired.
51	Roberts, R. I. (1997) [84]	Wales	Roberts, R. I. “The Welsh shared care prescribing project”	This article discusses the Welsh shared care prescribing project.	*N* article not available. The staff performing the systematic review were unable to obtain the full text of the article.
52	Sibbald, B., et al. (1992) [85]	England	Prescribing at the hospital-general practice interface. II: impact of hospital outpatient dispensing policies in England on general practitioners and hospital consultants	This article evaluates the impact on general practitioners and hospital consultants on outpatient dispensing policies.	I: Intervention not delivered by a pharmacist in general practice. The intervention was not delivered by the person specified in the review criteria.
53	Shemilt et al. (2021) [86]	England	An evaluation into the refusal of essential shared care agreements: quetiapine	The abstract evaluated the documented reasons for primary care refusal of prescribing quetiapine under ESCAs in one UK NHS Trust.	I: Intervention not delivered by a pharmacist in general practice. The intervention was not delivered by the person specified in the review criteria. C: Wrong design. The design of the study was not the one desired.
54	Smith, S. M., et al. (2017) [87]	International (Systematic Review: Cochrane Review). Authors based in Republic of Ireland	Shared care across the interface between primary and specialty care in the management of long-term conditions.	This systematic review examines the effectiveness of shared care health service interventions.	I: Intervention not delivered by a pharmacist in general practice. The intervention was not delivered by the person specified in the review criteria.
55	Sowerby, C. and D. Taylor (2017) [88]	England	Cross-sector user and provider perceptions on experiences of shared-care clozapine: a qualitative study	This article examines stakeholder perceptions on delivering a shared-care clozapine service and understanding its effectiveness and acceptability of this service.	I: Intervention not delivered by a pharmacist in general practice. The intervention was not delivered by the person specified in the review criteria.
56	Steckowych, K. and M. Smith (2018) [89]	USA	Lessons learned and real-world challenges of implementing clinical pharmacy services in a primary care office	Conference abstract on the implementation and challenges faced while starting new clinical pharmacy services within primary care.	C: Wrong design. The design of the study was not the one desired.
57	Swallow, V. M., et al. (2013) [90]	England	Multidisciplinary teams, and parents, negotiating common ground in shared care of children with long-term conditions: A mixed methods study	This article examines the multi-method study of social interaction between multidisciplinary teams and parents as they shared clinical care.	P: Wrong setting. The research setting was not correct. C: Wrong design. The design of the study was not the one desired.
58	Taylor, D., et al. (2010) [91]	England	User and staff perspectives of clozapine clinic services	The article examines the stakeholder’s perspectives of specialist clozapine clinics in England.	P: Wrong setting. Something about the research setting was not correct. I: Intervention not delivered by a pharmacist in general practice. The intervention was not delivered by the person specified in the review criteria.
59	Terry, D. R. P. (2011) [92]	England	Medicines management across the primary-hospital healthcare interface: a study of paediatric patients	This thesis examines medicines management across the primary and secondary interface for paediatric patients and how processes can be improved.	I: Intervention not delivered by a pharmacist in general practice. The intervention was not delivered by the person specified in the review criteria. C: Wrong design. The design of the study was not the one desired.
60	Terry, D., et al. (2012) [93]	England	Prescribing for children at the interfaces of care	This article reviews the current arrangements in England relating to prescribing for children at the interfaces of care.	C: This was a commentary.
61	Travis, S. S. and Bethea L. S. (2001) [94]	USA	Medication administration by family members of dependent elders in shared care arrangements	This article examines family member’s experiences with medication administration.	I: Intervention not delivered by a pharmacist in general practice. The intervention was not delivered by the person specified in the review criteria. C: Wrong design. The design of the study was not the one desired.
62	Tolley L. et al. (2021) [95]	England	An evaluation into the refusal of essential shared care agreements: aripiprazole	The abstract evaluated the documented reasons for primary care refusal of prescribing aripiprazole under ESCAs in one UK NHS Trust.	I: Intervention not delivered by a pharmacist in general practice. The intervention was not delivered by the person specified in the review criteria. C: Wrong design. The design of the study was not the one desired.
63	Walker, M. (2001) [96]	England	Shared care for opiate substance misusers in Berkshire	A discussion paper on shared care services for opiate substance misusers.	C: This was a commentary.
64	Yones, E., et al. (2019) [97]	England	Prescribing dronedarone for paroxysmal atrial fibrillation: how is it done across the UK and is it safe?	A short report on the prescribing of dronedarone in the UK and how it can be safely prescribed with a local shared care protocol.	I: Intervention not delivered by a pharmacist in general practice. The intervention was not delivered by the person specified in the review criteria.

Final Population, Intervention, Comparison, Outcome (PICO)-based taxonomy of reasons used to exclude articles from systematic reviews. *N* not a PICO-based exclusion reason

## Discussion

This paper aimed to systematically assess the pharmacist’s role in general practice in supporting the implementation of SCA in primary care. No articles met our inclusion criteria. Several studies have tried to define the role of pharmacists in general practice, but the working definition has not been defined by healthcare organisations or the research community [98, 99]. Another reason for there to be no studies available is because the GP pharmacist’s role is still a relatively new role in healthcare and little existing literature in role evolution is available [100].

### Comparison with other studies

There is an agreement in the literature that the pharmacist role is developing at pace within the general practice setting and recent international systematic reviews and meta-analyses demonstrate positive effects on medication use and clinical outcomes [99, 101]. Pharmacists integrated into general practice teams can perform a variety of roles. This includes direct patient care, medicines reconciliation and education to members of the healthcare team and in the detection and resolution of medication-related problems [102–104]. A recent review of the impact of integrating pharmacists into primary care teams on health systems indicators on healthcare utilisation by Hayhoe et al. did highlight the activities provided by pharmacists in general practice. Still, it failed to discover activities to support SCA in the published literature [105]. Internationally, other terminologies and contexts vary, giving one example being collaborative care agreements by Stuhec et al. in Slovenia [106–109]. The focus has been aimed in this international context as clinical pharmacists share a role within their own heterogeneous setting and sharing this with various physicians in managing a patient with chronic conditions, not necessarily in the same setting such as GP surgery in the UK where the GP is seen anecdotally as the main gatekeeper and first point of contact for all medication issues related to the patient. The observational studies by Stuhec et al. have focused on older patient and psychiatric patients. Further research would be required to homogenise and formalise an internationally recognised term for shared care agreements.

The most recent Cochrane systematic review, which assessed the effectiveness of shared care across the interface between primary and speciality care in the management of long-term conditions, did not consider models of integrating care between pharmacists and primary care physicians [18]. The authors of the Cochrane review stated that this limited its generalisability to all types of collaborative care due to the contextual specificity. The review did note that several other models of shared or collaborative care should be considered for review. This systematic review has highlighted that the model of care involving a pharmacist in primary care supporting SCA is not currently present in the literature and should be considered for further investigation.

### Implications of the review findings on clinicians and decision-makers in healthcare and future studies

The changing needs of the population make it increasingly important that the patient’s multiple needs are met in a well-coordinated way. To respond effectively to these changing needs, healthcare teams need to utilise the skills available across our healthcare teams. To help alleviate these pressures, it has been recommended that the NHS should maximise the opportunities offered by pharmacists [9, 110, 111]. Pharmacists can take on significant amounts of work currently done by GPs and other staff in general practice [111]. To explore whether shared care management could be implemented successfully by the pharmacist in general practice, we need to understand the relevant and important pharmacist related barriers and facilitators concerning the implementation of this role. The influence of these factors needs to be recognised and considered and how this can inform further research into this area.

### Decision-making

Collaboration between pharmacists and the healthcare team is of paramount importance, and understanding the skills of multiple healthcare professionals will help support an integrated system [112]. We need to understand the role in which pharmacists will support SCA and how general practitioners are willing to integrate pharmacists within this process. A finding from earlier research has shown that general practice has a positive experience of pharmacist recommendations in a range of conditions [113–115]. However, how would this apply to SCA when patients and healthcare staff need to understand the role of the pharmacist and agree to the SCA? Would patients accept pharmacists undertaking this new role within the general practice?

### Funding and workload

General practice continues to be concerned with the inappropriate funding associated with supporting shared care medication [21]. Commissioners have always stated that the funding of specialist’s drugs has been agreed so cost should not be an issue. General practitioners have expressed concerns that the additional workload required to support the integration of specialised medication is a factor they believe has not been appreciated [116]. This has been exacerbated by the COVID-19 pandemic, which has seen a rise in how general practice is expected to manage care which would typically be carried out in a hospital setting which in turn has contributed to a growing workload in primary care [117]. Previous studies have suggested that the impact of practice-based pharmacists will not be on workload but quality and safety [118]. It is reasonable to ask whether GPs would support pharmacists taking on this role without appropriate funding and whether this intervention will reduce workload in general practice.

### Working definition and liability

We need to consider a working definition of the practice pharmacist role in shared care. The differences in pharmacists’ primary care roles have been identified in the international literature. The lack of clarity and knowledge of this primary care role can negatively affect their potential integration into the primary care team [119]. The knowledge, skills and attitudes required to support SCA should be made readily available to practice pharmacists, primary care teams and the general public. This would enable SCA management to be developed and applied nationally across primary care. The rapid emergence of new professional roles for pharmacists also means that arrangement in respect of liability needs to keep up with the changing nature of pharmacy practice within this more complex intervention.

This empty review can act as a platform to inform policymakers in healthcare that there is a lack of robust evidence that evaluates the role and potential value of pharmacists supporting SCA in general practice. Healthcare systems must seek out the best possible evidence to support patients within this new healthcare environment. However, the absence of research in this area does not justify the rejection of this intervention [120]. As the role is relatively new, there is still work to be done to develop the evidence base of pharmacists working in general practice to support SCA and the benefits of this role within the healthcare system.

### Limitations

A limitation of this review is that the search strategy included a literature search of articles only in the English language. Other articles may have been published on pharmacists supporting SCA in general practice in non-English journals. Personal commentaries, blogs and opinion pieces were excluded from this systematic review due to the research design. This may have excluded observations that are occurring in general practice but have not been critically appraised. Another limitation is that the term SCA may be used as an alternate term from an international perspective. Despite the use of 12 healthcare-related bibliographical databases and the extensive use of keywords to maximise the sensitivity of relevant studies, after removal of duplicates, this yielded a low number of citations of titles and abstracts, another limitation. This systematic review used database word stock to establish standard search terms; however, it cannot be discounted that this may not have retrieved all articles relating to this intervention. Another limitation that needs to be considered is that abstracts from conference proceedings were not included in the synthesis, which could account for the empty return due to the high evidence bar for the systematic review.

## Conclusion

This systematic review identified no eligible studies on the interventions provided by a pharmacist in supporting SCA in general practice. It is not possible to formulate what the role pharmacists can play in supporting SCA in general practice based on scientific evidence. There is an urgent need for studies that identify, observe and evaluate GP-based pharmacists’ roles concerning SCA that currently occur in clinical practice. Comparing and contrasting each general practice’s approach will ensure the development of a consensus for the role of GP pharmacists on SCA based on the current SCAs occurring in general practice, and how to implement this intervention consistently. The role of the pharmacist is expanding in general practice, and interventions which prove beneficial for patients and the healthcare system are required to meet the ever-changing demand in healthcare and to ensure that these new interventions follow the evidence. This empty systematic review serves as a starting point for further clinical research in this area.

## Supplementary Information

**Additional file 1: S1.** List of Search Terms.

**Additional file 2: S2.** The Preferred Reporting Items for Systematic Reviews and Meta-Analysis (PRISMA) checklist.

**Additional file 3: S3.** PRISMA 2020 for Abstracts Checklist.

## Data Availability

We provide all supporting data in the manuscript in Fig. 1, and Table 1, Search Strategy S1, PRISMA statement (S2) and abstract checklist (S3).

## Abbreviations

SCA: Shared care agreement
HRQoL: Health-related quality of life
MeSH: Medical Subject Heading terms
LTC: Long-term conditions
NHS: National Health Service
BMA: British Medical Association
PRISMA: Preferred Reporting Items for Systematic Review and Meta-Analysis
MMAT: Mixed Method Appraisal Tool
