# Supplementary material for: Systematic literature review of pharmacists in general practice in supporting the implementation of shared care agreements in primary care
Source: Syst Rev. 2022 May 11;11:88. doi: 10.1186/s13643-022-01933-4 (PMC9091138; doi:10.1186/s13643-022-01933-4)
Supplement: Supplementary file 1 — Additional file 1: S1. List of Search Terms. [file 13643_2022_1933_MOESM1_ESM.docx]

**S1 List of Search Terms**

**Search terms for AMED® (1985 to 07.11.2021), Platform: Ovid®.**

1. (Family Practice or General Practice).af.
2. (Physicians, Family or General Practitioners).af.
3. general pract:.af.
4. MH general practitioner.af.
5. family medicine.af.
6. family Health.af.
7. primary Health Care.af.
8. comprehensive health care.af.
9. MH health care.af
10. (primary care or primary health care or primary healthcare).af.
11. MH primary medical care.af.
12. GP.af.
13. 1 or 2 or 3 or 4 or 5 or 6 or 7 or 8 or 9 or 10 or 11 or 12
14. Secondary Care.af.
15. (secondary care or secondary healthcare or secondary health care).af.
16. Hospitals.af.
17. MH hospital.af.
18. Inpatients.af.
19. MH hospital patient.af.
20. MH outpatient.af.
21. Consultants.af.
22. Physician:.af.
23. consultant:.af.
24. (specialist or specialists).af.
25. MH medical specialist.af.
26. MH Tertiary Healthcare.af.
27. 14 or 15 or 16 or 17 or 18 or 19 or 20 or 21 or 22 or 23 or 24 or 25 or 26
28. 13 or 27
29. (interfac: or share: or SCA).af.
30. (Pharmacis: or non-dispensin: or Clinical Pharmacis:).af.
31. 28 and 29 and 30

**Retrieved 5**

**Search terms for Database CINAHL® (1950 to 07.11.2021), Platform: EBSCO®.**

1. S1 MH Family Practice OR MH Physicians, Health
2. S2 general pract*
3. S3 Family medicine
4. S4 MH Family Health
5. S5 family health
6. S6 MH Primary Health Care OR MH Health care
7. S7 primary care OR primary health care OR primary healthcare OR MH primary medical care
8. S8 GP
9. S9 S1 OR S2 OR S3 OR S4 OR S5 OR S6 OR S7 OR S8
10. S10 MH Secondary Health Care
11. S11 secondary care OR secondary health care OR secondary healthcare
12. S12 MH Hospitals
13. S13 MH Inpatients OR MH Outpatient MH Tertiary care
14. S14 MH Consultants
15. S15 MH Physicians
16. S16 consultant* OR specialist OR specialists OR MH medical specialist
17. S17 S10 OR S11 OR S12 OR S13 OR S14 OR S15 OR S16
18. S18 S9 OR S17
19. S19 interfac* OR share* OR SCA
20. S20 Pharmacis* OR non-dispensin* OR Clinical Pharmacis*
21. S21 S18 AND S19 AND S20

**Retrieved 238**

**Search terms for Cochrane library, (accessed on 07.11.2021) Platform Wiley® online library**

(“family practice” OR “family physician*” OR “general pract*” OR “family medicine” OR “family health” OR “family healthcare” OR “primary health care” OR “primary care” OR “primary healthcare” OR " primary medical care" OR "health care" GP OR “secondary health care” OR “secondary care” OR “secondary healthcare” OR hospital* OR inpatient* OR consultant* OR physician* OR consultant* OR specialist* OR medical specialist OR tertiary care) AND (interfac* OR share* OR SCA) AND (pharmacist* OR non-dispensing* OR “clinical pharmacist*”)

**Retrieved 280**

**Search terms for Database Embase® (1974 to 07.11.2021), Platform: Ovid®.**

1. (Family Practice or General Practice).af.
2. (Physicians, Family or General Practitioners).af.
3. general pract:.af.
4. MH general practitioner.af.
5. family medicine.af.
6. family Health.af.
7. primary Health Care.af.
8. comprehensive health care.af.
9. MH health care.af
10. (primary care or primary health care or primary healthcare).af.
11. MH primary medical care.af.
12. GP.af.
13. 1 or 2 or 3 or 4 or 5 or 6 or 7 or 8 or 9 or 10 or 11 or 12
14. Secondary Care.af.
15. (secondary care or secondary healthcare or secondary health care).af.
16. Hospitals.af.
17. MH hospital.af.
18. Inpatients.af.
19. MH hospital patient.af.
20. MH outpatient.af.
21. Consultants.af.
22. Physician:.af.
23. consultant:.af.
24. (specialist or specialists).af.
25. MH medical specialist.af.
26. MH Tertiary Healthcare.af.
27. 14 or 15 or 16 or 17 or 18 or 19 or 20 or 21 or 22 or 23 or 24 or 25 or 26
28. 13 or 27
29. (interfac: or share: or SCA).af.
30. (Pharmacis: or non-dispensin: or Clinical Pharmacis:).af.
31. 28 and 29 and 30

**Retrieved 2570**

**Search terms Emcare® (1995 to 07.11.2021), Platform: Ovid®.**

1. (Family Practice or General Practice).af.
2. (Physicians, Family or General Practitioners).af.
3. general pract:.af.
4. MH general practitioner.af.
5. family medicine.af.
6. family Health.af.
7. primary Health Care.af.
8. comprehensive health care.af.
9. MH health care.af
10. (primary care or primary health care or primary healthcare).af.
11. MH primary medical care.af.
12. GP.af.
13. 1 or 2 or 3 or 4 or 5 or 6 or 7 or 8 or 9 or 10 or 11 or 12
14. Secondary Care.af.
15. (secondary care or secondary healthcare or secondary health care).af.
16. Hospitals.af.
17. MH hospital.af.
18. Inpatients.af.
19. MH hospital patient.af.
20. MH outpatient.af.
21. Consultants.af.
22. Physician:.af.
23. consultant:.af.
24. (specialist or specialists).af.
25. MH medical specialist.af.
26. MH Tertiary Healthcare.af.
27. 14 or 15 or 16 or 17 or 18 or 19 or 20 or 21 or 22 or 23 or 24 or 25 or 26
28. 13 or 27
29. (interfac: or share: or SCA).af.
30. (Pharmacis: or non-dispensin: or Clinical Pharmacis:).af.
31. 28 and 29 and 30

**Retrieved 782**

**Search terms for Google Scholar® (07.11.2021) Platform: Google UK®**

(pharmacy or pharmacist) AND "shared care" AND ("primary care" or "Secondary care") AND "interface"

**Retrieved 607**

**Search terms for Database Healthcare Management Information Consortium (HMIC®) (1979 to November 2021), Platform: Ovid®.**

1. (Family Practice or General Practice).af.
2. (Physicians, Family or General Practitioners).af.
3. general pract:.af.
4. MH general practitioner.af.
5. family medicine.af.
6. family Health.af.
7. primary Health Care.af.
8. comprehensive health care.af.
9. MH health care.af
10. (primary care or primary health care or primary healthcare).af.
11. MH primary medical care.af.
12. GP.af.
13. 1 or 2 or 3 or 4 or 5 or 6 or 7 or 8 or 9 or 10 or 11 or 12
14. Secondary Care.af.
15. (secondary care or secondary healthcare or secondary health care).af.
16. Hospitals.af.
17. MH hospital.af.
18. Inpatients.af.
19. MH hospital patient.af.
20. MH outpatient.af.
21. Consultants.af.
22. Physician:.af.
23. consultant:.af.
24. (specialist or specialists).af.
25. MH medical specialist.af.
26. MH Tertiary Healthcare.af.
27. 14 or 15 or 16 or 17 or 18 or 19 or 20 or 21 or 22 or 23 or 24 or 25 or 26
28. 13 or 27
29. (interfac: or share: or SCA).af.
30. (Pharmacis: or non-dispensin: or Clinical Pharmacis:).af.
31. 28 and 29 and 30

**Retrieved 78 articles**

**Search terms for Database MEDLINE® (1946 to 07.11.2021), Platform: Ovid®.**

1. (Family Practice or General Practice).af.
2. (Physicians, Family or General Practitioners).af.
3. general pract:.af.
4. MH general practitioner.af.
5. family medicine.af.
6. family Health.af.
7. primary Health Care.af.
8. comprehensive health care.af.
9. MH health care.af
10. (primary care or primary health care or primary healthcare).af.
11. MH primary medical care.af.
12. GP.af.
13. 1 or 2 or 3 or 4 or 5 or 6 or 7 or 8 or 9 or 10 or 11 or 12
14. Secondary Care.af.
15. (secondary care or secondary healthcare or secondary health care).af.
16. Hospitals.af.
17. MH hospital.af.
18. Inpatients.af.
19. MH hospital patient.af.
20. MH outpatient.af.
21. Consultants.af.
22. Physician:.af.
23. consultant:.af.
24. (specialist or specialists).af.
25. MH medical specialist.af.
26. MH Tertiary Healthcare.af.
27. 14 or 15 or 16 or 17 or 18 or 19 or 20 or 21 or 22 or 23 or 24 or 25 or 26
28. 13 or 27
29. (interfac: or share: or SCA).af.
30. (Pharmacis: or non-dispensin: or Clinical Pharmacis:).af.
31. 28 and 29 and 30

**Retrieved 856**

**Grey literature search**

**OpenSIGLE (opensigle.inist.fr accessed 7th November 2021)**

"primary care" OR "secondary care" OR "tertiary care” AND "pharmacist" AND "shared care"

**This led to 1336 article**

**Search terms for Database PsycINFO®, Psychology and Behavioural Sciences Collection, Health Business Elite, Biomedica Reference Collection: Comprehensive Library, Information Science & Technology Abstracts (1967 to 07.11.2021), Platform: ProQuest®.**

1. S1 Family Practice
2. S2 Physicians, Family
3. S3 general pract*
4. S4 Family medicine
5. S5 Family Health
6. S6 primary health care
7. S7 primary care
8. S8 primary medical care
9. S9 primary healthcare
10. S10 health care
11. S11 GP
12. S12 S1 OR S2 OR S3 OR S4 OR S5 OR S6 OR S7 OR S8 OR S9 OR S10 OR S11
13. S13 secondary health care
14. S14 secondary care
15. S15 secondary healthcare
16. S16 hospital*
17. S17 Inpatients
18. S18 outpatient
19. S19 Consultant*
20. S20 Physician*
21. S21 specialist*
22. S22 medical specialist
23. S23 tertiary*
24. S24 S13 OR S14 OR S15 OR S16 OR S17 OR S18 OR S19 OR S20 OR S21 OR S22 OR S23
25. S25 S12 OR S24
26. S26 interfac*
27. S27 share*
28. S28 SCA
29. S29 S26 OR S27 OR S28
30. S30 Pharmacis* OR non-dispensing* OR (Clinical Pharmacis*)
31. S31 S29 AND S30
32. S32 S31 AND S25

**Retrieved 85**

**Search terms for Scopus (2004 to 7.11.2021) Platform: Elsevier**

TITLE-ABS-KEY ( "family practice" OR "family physician*" OR "general pract*" OR "family medicine" OR "family health" OR "family healthcare" OR "primary health care" OR "primary care" OR "primary healthcare" OR "primary medical care" OR "GP" OR "health care" OR "secondary health care" OR "secondary care" OR "secondary health care" OR "secondary healthcare" OR "hospital*" OR "outpatient" OR "inpatient*" OR "consultant*" OR "physician*" OR "consultant*" OR "specialist*" OR "tertiary*" ) AND TITLE-ABS-KEY ( "interfac*" OR "share*" OR "SCA" ) AND TITLE-ABS-KEY ( "pharmacist*" OR "non-dispensing*" OR "clinical pharmacist*" )

**Retrieved 1,416**

**Search terms for Web of Science® Core Collection 1970-07.11.2021 Platform:** **Clarivate Analytics®**

“family Practice” OR “physicians, family” OR “general pract*” OR “family medicine” OR “family Health” OR “family healthcare” OR “primary health care” OR “primary care” OR “primary healthcare” OR “MH primary medical care“ OR “MH health care” OR “GP”

OR

“secondary health care” OR “secondary care” OR “secondary health care” OR “secondary healthcare” OR “hospital*” OR “inpatient*” or “MH outpatient” OR “consultant*” OR “physician*” OR “consultant*” OR “specialist*” OR “tertiary*”

AND

“interfac*” OR “share*” OR “SCA”

AND

“Pharmacis*” OR “non-dispensing*” OR “Clinical Pharmacis*”

**Retrieved 810**
